# Dose adjustment of intravitreal medications and gases according to axial length and vitreous cavity volume

**DOI:** 10.5935/0004-2749.2025-0077

**Published:** 2025-09-10

**Authors:** Rodrigo Pessoa Cavalcanti Lira, Ana Paula Teles Silveira, Gabriel Rocha Lira, Maria Isabel Lynch Gaete

**Affiliations:** 1 Universidade Federal de Pernambuco, Recife, PE, Brazil

**Keywords:** Intravitreal injections, Axial length, Vitreous body, Drug dosage calculations, Pharmacokinetics, Anti-infective agents

## Abstract

**Purpose:**

Standard intravitreal medication dosages are based on an assumed vitreous cavity volume of 4.0-4.5 mL. However, individual variations in vitreous cavity volume may influence both the efficacy and safety of these medications. This study proposes dosage adjustments for intravitreal medications and gases according to axial length and the corresponding vitreous cavity volume.

**Methods:**

This descriptive study employed reference guidelines that use axial length to estimate the Axial Length-based Volume of the Vitrectomized Space and the Vitreous Volume EXact table for determining dose adjustments across varying eye sizes. Small eyes (axial length 19-22 mm) have an average vitreous cavity volume of 3.5 mL at an axial length of 20.5 mm; standard-sized eyes (22-25 mm) have 4.8 mL at 23.5 mm; large eyes (25-28 mm) have 6.4 mL at 26.5 mm; and extra-large eyes (28-32 mm) have 8.4 mL at 29.5 mm. The medications considered included anti-infectives, anti-VEGFs, complement inhibitors, recombinant proteases, chemotherapy agents, corticosteroids, and medical gases.

**Results:**

Analysis of intravitreal drug concentrations relative to vitreous cavity volume demonstrated notable variability when a standard dose was administered. Small eyes received about 135% of the concentration intended for a standard-sized eye; large eyes received around 75%; and extra-large eyes received under 60%. The recommended dose adjustments are as follows: for small eyes, administer 70-80% of the standard dose; for large eyes, 130-140%; and for extra-large eyes, 170-180%.

**Conclusions:**

Tailoring intravitreal drug and gas dosages according to axial length and vitreous cavity volume may enhance intraocular drug distribution, potentially improving both safety and therapeutic outcomes.

## INTRODUCTION

The dimensions of the vitreous cavity vary considerably among individuals^(1^-^4)^. Despite this, the standardized dosages provided in package inserts and treatment protocols for intravitreal medications are still based on an assumed fixed vitreous cavity volume (VCV) of 4.0-4.5 mL. As a result, both myopic and hyperopic eyes receive the same doses as average-sized eyes.

Intravitreal therapy has the distinct benefit of delivering localized treatment for intraocular diseases, with ongoing advancements in drugs and therapeutic approa ches^(4)^. Paracelsus’ well-known phrase, “Sola dosis facit venenum” (“the dose makes the poison”), underscores that any substance can be harmful if administered in excessive amounts. In numerous medical fields-including pediatrics, intensive care, oncology, and anesthesia-drug dosages are tailored according to parameters such as body weight or body mass index. However, ophthalmology generally continues to apply fixed intravitreal doses under the assumption that all eyes are the same size. This approach stands in contrast to several studies demonstrating variation in ocular volume^(5^,^6)^. To prevent intravitreal medications from reaching toxic levels, it is necessary to adjust dosages based on the actual vitreous volume, as supported by recent research^(7^,^8)^. Intravitreous injections (IVI) that do not result in drug reflux leads to increased intraocular pressure (IOP). Koçak et al.^(9)^ reported a significant inverse correlation between VCV and post-IVI IOP elevation. Although similar IOP increases were noted in eyes with low and medium VCV, the rise was less substantial in eyes with larger VCV.

A recent study, Vitreous Volume EXact (VIVEX)^(3)^, introduced a table for estimating VCV based on axial length (AL). However, there are several valid criticisms. The VIVEX table was derived from a retrospective observational study utilizing magnetic resonance imaging data from only 72 eyes, with ALs ranging from 20.47 to 30.55 mm. The data distribution was uneven, with approximately 85% of the eyes falling between 21 and 26.5 mm. Furthermore, the table provides estimates for eyes with ALs between 18 and 30 mm, thereby extrapolating beyond the lower boundary of the AL range actually analyzed.

The space formed within the vitreous cavity following vitrectomy—referred to as the vitrectomized space (VVS)—shows a strong correlation with the eye’s AL^(2^,^10)^. The Axial Length-based Volume of the Vitrectomized Space (ALVIS) study^(4)^ offered a method for estimating VVS by categorizing individuals according to AL, sex, and cataract surgery history. This cross-sectional observational study included 144 randomly selected vitrectomized eyes, with ALs ranging from 20 to 32 mm. A strong positive correlation between AL and VVS was reported (r=0.968; p<0.001). This relationship held true across sexes and in both phakic and pseudophakic eyes. The study concluded by establishing a guideline for estimating VVS from AL using a cubic polynomial regression model.

The currently used doses are regarded as safe and effective for the majority of eyes. However, their safety in small or large eyes cannot be confirmed, as specific studies addressing this issue are lacking. In this context, the present study proposes individualized dosing guidelines for different intravitreal medications and gases, based on the calculated VCV for small, large, and extra-large eyes as determined by AL measurements, with the goal of enhancing treatment precision and efficacy.

## METHODS

This descriptive study was carried out in 2025 by the Department of Ophthalmology at the Federal University of Pernambuco, Brazil, and was based on data obtained from the literature.

Reference frameworks included the ALVIS^(4)^ guidelines and the VIVEX table^(3)^, which were used to guide dose adjustments for small, large, and extra-large eyes.

- Small eyes were defined as having an AL between 19 and 22 mm.
- Standard-sized eyes had an AL between 22 and 25 mm.
- Large eyes had an AL between 25 and 28 mm.
- Extra-large eyes had an AL between 28 and 32 mm.

**Figure 1:**
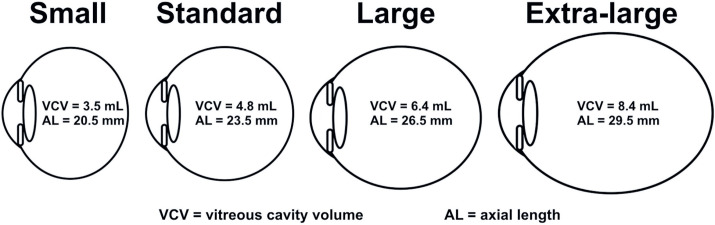
Categorization of eye size based on axial length and vitreous cavity volume

To estimate medication concentration and the percentage of the standard dose retained in the vitreous cavity based on VCV, the following average VCV values were applied (Figure 1):

- Small eyes: 3.5 mL (corresponding to an AL of 20.5 mm)
- Standard-sized eyes: 4.8 mL (corresponding to an AL of 23.5 mm)
- Large eyes: 6.4 mL (corresponding to an AL of 26.5 mm)
- Extra-large eyes: 8.4 mL (corresponding to an AL of 29.5 mm)

The medications and medical gases considered in this study included the following: anti-infective agents (amphotericin-B, amikacin, ceftazidime, ciprofloxacin, clindamycin, foscarnet, ganciclovir, gentamicin, moxifloxacin, vancomycin, and voriconazole), anti-VEGF agents, complement inhibitors, and recombinant protea ses (aflibercept 2 mg, aflibercept 8 mg, brolucizumab, faricimab, ocriplasmin, pegcetacoplan, ranibizumab 0.3 mg, and ranibizumab 0.5 mg), chemotherapy (methotrexate), corticosteroids (dexamethasone sodium phosphate, triamcinolone), and medical gases (octafluoropropane and sulfur hexafluoride).

Dose adjustments were proposed based on AL for small, large, and extra-large eyes to ensure that the concentrations of medications or medical gases remained comparable to those in standard-sized eyes. For intravitreal medications, a concentration difference of up to 2% was accepted in small, large, and extra-large eyes. In the case of intravitreal gases, a difference of up to 6% was tolerated in small and large eye and up to 13% in extra-large eyes.

Syringe sizes were recommended according to the volume to be administered:

- Syringes with capacities of 0.3, 0.5, 1, 3, and 20 mL feature graduation marks at 0.1 mL intervals.
- Syringes with a 60 mL capacity have graduation marks at 0.2 mL intervals.

Ethical committee review was not required, as outlined in Article 1 of Resolution 510/2016. According to this regulation, certain types of research are exempt from registration or evaluation by the CEP/CONEP (National Council of Ethics and Research-Brazil), including research that uses publicly available information, in accordance with Law No. 12.527 (Brazil), dated November 18, 2011, research utilizing public domain data, research involving databases with aggregated information that does not allow individual identification, and research aimed at the theoretical exploration of situations arising spontaneously and contingently in professional practice, provided that identifying information is not disclosed^(11)^.

## RESULTS

When considering the VCV, analysis of medication concentrations in the vitreous humor after administration of a standard dose showed notable differences (Table 1):

**Table 1:** Medication concentration and percentage of the recommended dose in the vitreous following administration of the standard dose, relative to vitreous cavity volume and axial length

Medication	Dose	Volume	Concentration in the syringe	Concentration in the vitreous cavity						
				Small eye	PRD	Standard eye	Large Eye	PRD	Extra-large eye	PRD
				AL=20.5mm-VCV≅3.5mL		AL=23.5mm-VCV≅4.8mL	AL=26.5mm-VCV≅6.4mL		AL=29.5 mm-VCV≅8.4mL	
**Anti-infective**	**mg**	**mL**	**mg/mL**	**mg/mL**	**%**	**mg/mL**	**mg/mL**	**%**	**mg/mL**	**%**
Amphotericin B	0.005	0.1	0.05	0.0014	136	0.001	0.0008	75	0.0006	58
Amikacin	0.4	0.1	4	0.11	136	0.08	0.06	75	0.05	58
Ceftazidime	2.25	0.1	23	0.63	136	0.46	0.35	75	0.26	58
Ciprofloxacin	0.05	0.05	1	0.014	137	0.01	0.008	75	0.006	57
Clindamycin	10.1	0.1	101	2.81	136	2.06	1.55	75	1.19	58
Foscarnet	2.4	0.1	24	0.67	136	0.49	0.37	75	0.28	58
Ganciclovir	6	0.1	60	1.67	136	1.22	0.92	75	0.71	58
Gentamicin	0.2	0.1	2	0.06	136	0.04	0.03	75	0.02	58
Moxifloxacin	0.1	0.05	2	0.03	137	0.02	0.02	75	0.01	57
Vancomycin	1	0.1	10	0.28	136	0.2	0.15	75	0.12	58
Voriconazole	0.05	0.05	1	0.014	137	0.01	0.008	75	0.006	57
**Anti-VEGFs, complement inhibitors, and recombinant proteases**	**mg**	**mL**	**mg/mL**	**mg/mL**	**%**	**mg/mL**	**mg/mL**	**%**	**mg/mL**	**%**
Aflibercept 2 mg	2	0.05	40	0.56	137	0.41	0.31	75	0.24	57
Aflibercept 8 mg	8	0.07	114	2.24	136	1.64	1.24	75	0.94	57
Bevacizumab	1.25	0.05	25	0.35	137	0.26	0.19	75	0.15	57
Brolucizumab	6	0.05	120	1.69	137	1.24	0.93	75	0.71	57
Faricimab	6	0.05	120	1.69	137	1.24	0.93	75	0.71	57
Ocriplasmin	0.125	0.1	1.3	0.03	136	0.03	0.02	75	0.01	58
Pegcetacoplan	15	0.1	150	4.17	136	3.06	2.31	75	1.76	58
Ranibizumab 0.3 mg	0.3	0.05	6	0.08	137	0.06	0.05	75	0.04	57
Ranibizumab 0.5 mg	0.5	0.05	10	0.14	137	0.1	0.08	75	0.06	57
**Chemotherapy**	**mg**	**mL**	**mg/mL**	**mg/mL**	**%**	**mg/mL**	**mg/mL**	**%**	**mg/mL**	**%**
Methotrexate	0.4	0.1	4	0.11	136	0.08	0.06	75	0.05	58
**Corticosteroids**	**mg**	**mL**	**mg/mL**	**mg/mL**	**%**	**mg/mL**	**mg/mL**	**%**	**mg/mL**	**%**
Dexamethasone	0.4	0.1	4	0.11	136	0.08	0.06	75	0.05	58
Triamcinolone	4	0.1	40	1.11	136	0.82	0.62	75	0.47	58
**Medical gases**	**Gas+air (mL)**	**mL**	**%**	**%**	**%**	**%**	**%**	**%**	**%**	**%**
Octafluoropropane	pure gas	0.7	100	16.67	131	12.73	9.86	77	7.69	60
Octafluoropropane	3+17	20	15	12.77	106	12.1	11.36	94	10.56	87
Octafluoropropane	8+52	60	13.3	12.6	102	12.35	12.05	98	11.7	95
Sulfur hexafluoride	pure gas	1.2	100	25.53	128	20	15.79	79	12.5	63
Sulfur hexafluoride	5+15	20	25	21.28	106	20.16	18.94	94	17.61	87
Sulfur hexafluoride	12+48	60	20	18.9	102	18.52	18.07	98	17.54	95

PRD= percentage of the recommended dose; AL= axial length; VCV= vitreous cavity volume.

- Small eyes received approximately 135% of the dose recommended for a standard-sized eye.
- Large eyes received only 75% of the recommended dose.
- Extra-large eyes received less than 60% of the recommended dose.

Based on these findings, dose adjustments for intravitreal medications and medical gases according to the eye’s AL and VCV are proposed as follows (Table 2):

**Table 2:** Suggested dose adjustments according to axial length

Medications	Concentration in the syringe	Size of the syringe	Volume to be injected						
			Small eye	PRD	Standard eye	Large eye	PRD	Extra-large eye	PRD
			19<AL≤22mm		22<AL≤25mm	25<AL≤28mm		28<AL≤31mm	
**Anti-infective**	**mg/mL**	**mL**	**mL**	**%**	**mL**	**mL**	**%**	**mL**	**%**
Amphotericin B	0.05	0.3 or 0.5	0.07	101	0.10	0.13	100	0.17	99
Amikacin	4	0.3 or 0.5	0.07	101	0.10	0.13	100	0.17	99
Ceftazidime	23	0.3 or 0.5	0.07	101	0.10	0.13	100	0.17	99
Ciprofloxacin	1	0.3 or 0.5	0.04	100	0.05	0.07	100	0.09	100
Clindamycin	101	0.3 or 0.5	0.07	101	0.10	0.13	100	0.17	99
Foscarnet	24	0.3 or 0.5	0.07	101	0.10	0.13	100	0.17	99
Ganciclovir	60	0.3 or 0.5	0.07	101	0.10	0.13	100	0.17	99
Gentamicin	2	0.3 or 0.5	0.07	101	0.10	0.13	100	0.17	99
Moxifloxacin	2	0.3 or 0.5	0.04	100	0.05	0.07	100	0.09	100
Vancomycin	10	0.3 or 0.5	0.07	101	0.10	0.13	100	0.17	99
Voriconazole	1	0.3 or 0.5	0.04	100	0.05	0.07	100	0.09	100
**Anti-VEGFs, complement inhibitors, and recombinant proteases**	**mg/mL**	**mL**	**mL**	**%**	**mL**	**mL**	**%**	**mL**	**%**
Aflibercept 2 mg	40	0.3 or 0.5	0.04	100	0.05	0.07	100	0.09	100
Aflibercept 8 mg	114	0.3 or 0.5	0.05	101	0.07	0.09	100	0.12	99
Bevacizumab	25	0.3 or 0.5	0.04	100	0.05	0.07	100	0.09	100
Brolucizumab	120	0.3 or 0.5	0.04	100	0.05	0.07	100	0.09	100
Faricimab	120	0.3 or 0.5	0.04	100	0.05	0.07	100	0.09	100
Ocriplasmin	1.3	0.3 or 0.5	0.07	101	0.10	0.13	100	0.17	99
Pegcetacoplan	150	0.3 or 0.5	0.07	101	0.10	0.13	100	0.17	99
Ranibizumab 0.3 mg	6	0.3 or 0.5	0.04	100	0.05	0.07	100	0.09	100
Ranibizumab 0.5 mg	10	0.3 or 0.5	0.04	100	0.05	0.07	100	0.09	100
**Chemotherapy**	**mg/mL**	**mL**	**mL**	**%**	**mL**	**mL**	**%**	**mL**	**%**
Methotrexate	4	0.3 or 0.5	0.07	101	0.10	0.13	100	0.17	99
**Corticosteroids**	**mg/mL**	**mL**	**mL**	**%**	**mL**	**mL**	**%**	**mL**	**%**
Dexamethasone	4	0.3 or 0.5	0.07	101	0.10	0.13	100	0.17	99
Triamcinolone	40	0.3 or 0.5	0.07	101	0.10	0.13	100	0.17	99
**Medical gases**	**Gas+air (mL)**	**mL**	**mL**	**%**	**mL**	**mL**	**%**	**mL**	**%**
Octafluoropropane	Pure gas	1^A^ or 3	0.5	104	0.7	0.9	97	1.2	95
Octafluoropropane	3 gas+17 air	20	20.0	106	20.0	20.0	94	20.0	87
Octafluoropropane	8 gas+52 air	60	60.0	102	60.0	60.0	98	60.0	95
Sulfur hexafluoride	Pure gas	1^B^ or 3	0.9	106	1.2	1.5	96	1.9	93
Sulfur hexafluoride	5 gas+15 air	20	20.0	106	20.0	20.0	94	20.0	87
Sulfur hexafluoride	12 gas+48 air	60	60.0	102	60.0	60.0	98	60.0	95

PRD= percentage of the recommended; AL= axial length; ^A^ not eligible for extra-large eyes; ^B^ only for small eyes.

- Small eyes: Inject 70-80% of the standard dose.
- Large eyes: Inject 130-140% of the standard dose.
- Extra-large eyes: Inject 170-180% of the standard dose.

## DISCUSSION

The current intravitreal medication doses are regarded as safe and effective for most patients. However, there is a lack of sufficient evidence to verify their safety in patients with either small or large eyes due to the absence of specific studies. To enhance the accuracy and effectiveness of treatment, this study suggests adjusting intravitreal medication doses based on the eye’s AL. Our proposed personalized dosing recommendations for various intravitreal medications and gases, calculated according to VCV derived from AL measurements, showed a dose variation more than 100% between small and extra-large eyes. This highlights the important need to address this frequently overlooked issue.

Regarding anti-VEGFs, complement inhibitors, and recombinant proteases, there is currently no reliable evidence to suggest that the number of drug-binding receptors on target tissues varies with eye size. A broad therapeutic range may exist that produces the same treatment effect. It is possible that the doses given are adequate for larger eyes while remaining safe for smaller eyes. Reibaldi et al.^(12)^ performed a meta-analysis of 52 randomized clinical trials evaluating the relationship between the intensity of anti-VEGF treatment and mortality risk and found no statistically significant association between treatment intensity and mortality. The dosing range for intravitreal anti-VEGF drugs—including ranibizumab, aflibercept, bevacizumab, and brolucizumab—is generally based on clinical trials and clinical experience. Anti-VEGF agents are commonly used to treat myopic choroidal neovascularization (mCNV), typically occurring in large or extra-large eyes^(13)^. Studies such as Zhu et al.^(14)^ support the effectiveness of these standard doses but stress the importance of tailoring treatment to individual patient characteristics to better control mCNV progression and potentially improve long-term visual outcomes. One of the few on-label dose adjustments is recommended for anti-VEGF treatment in retinopathy of prematurity (ROP). This adjustment is warranted due to the smaller size of the ocular globe and vitreous cavity in this population. Recent studies have examined the efficacy of varying antiangiogenic doses for treating ROP. Han et al.^(15)^ compared low and conventional doses of bevacizumab and reported promising results with the reduced doses. Similarly, Hillier et al.^(16)^ documented successful outcomes using ultralow doses of bevacizumab, indicating a possible decrease in toxicity without loss of efficacy. Additionally, Zhou et al.^(17)^ conducted network meta-analyses to evaluate success rates across different anti-VEGF doses and concluded that markedly lower doses can be effective. This variation in dosing and outcomes highlights the importance of a personalized approach that takes into account eye size and other individual patient factors.

Regarding anti-infectives, the minimum inhibitory concentration (MIC) is defined as the lowest concentration of an antibiotic needed to prevent bacterial growth after a specified incubation period. The MIC is essential for selecting the appropriate antibiotic and determining the correct dose for treating an infection. Moore et al.^(18)^ highlight the significance of the relationship between the peak antibiotic concentration and the MIC in achieving clinical efficacy of antibiotic therapy^(19)^. The safe dosage range of an antibiotic lies between the minimum effective dose and the maximum tolerated dose, which is determined through pharmacokinetic and pharmacodynamic studies that evaluate the drug’s absorption, distribution, metabolism, excretion, as well as its efficacy and toxicity^(19)^. Administering doses that are sublethal to bacteria may contribute to the development of antibiotic resistance, as noted by Andersson and Hughes^(20)^. For instance, in treating endophthalmitis, the recommended vancomycin dose is 1 mg/0.1 mL, while higher doses are toxic to the retina and can lead to complications such as hemorrhagic occlusive retinal vasculitis, which may cause severe vision loss. Pflugfelder et al.^(21)^ studied the retinal toxicity of intravitreal vancomycin, underscoring the risks linked to elevated doses. Vancomycin acts by inhibiting bacterial cell wall synthesis, but its toxicity may result from excessive accumulation in ocular tissues. Gan et al.^(22)^ studied the intravitreal levels of vancomycin and gentamicin in patients with postoperative endophthalmitis, highlighting the importance of using appropriate dosages to reduce toxicity. Ferro Desideri et al.^(8)^ presented evidence-based dosing guidelines for intravitreal medications in eyes filled with silicone oil, stressing the necessity of dose adjustments to prevent toxicity. Borkenstein et al.^(7)^ addressed the calculation of drug concentrations in intravitreal treatments, emphasizing the significance of determining dilution factors and accounting for deviations from recommended doses to avoid adverse effects.

Regarding chemotherapy, intravitreal methotrexate is used to treat various ocular disorders such as uveitis and intraocular lymphoma, typically at a dose of 400 µg/0.1 mL. Intravitreal delivery enables a high local concentration while reducing systemic side effects. McAllister et al.^(23)^ reviewed intravitreal methotrexate’s use in preventing and treating proliferative vitreoretinopathy, highlighting its efficacy at specific doses for each condition. However, higher methotrexate doses may cause ocular toxicity. Zhou et al.^(24)^ proposed a protocol for intravitreal methotrexate injections in treating primary vitreoretinal lymphoma, stressing the importance of careful monitoring to avoid toxicity. Vishnevskia-Dai et al.^(25)^ addressed potential toxic effects of elevated methotrexate doses in ocular leukemia manifestations, emphasizing the need to adjust doses based on eye size and patient response. Batchelor et al.^(26)^ reported on high-dose methotrexate for intrao cular lymphoma, highlighting the necessity of dose adjustments to prevent toxicity. In summary, intravitreal methotrexate administration should take into account eye volume and clinical condition to maximize efficacy and reduce toxicity risks.

Regarding corticosteroids, intravitreal triamcinolone acetonide is employed to treat several ocular conditions, including diabetic macular edema (DME), uveitic macular edema, and macular edema secondary to retinal vein occlusion (RVO). Notably, Bae et al.^(27)^ evaluated the dose-dependent effects of intravitreal triamcinolone on diffuse DME and found that doses between 4 mg and 8 mg were effective, with higher doses resulting in greater reductions in macular thickness. However, increased doses also raised the risk of side effects such as elevated IOP and cataract development. Similarly, the SCORE study by Scott et al.^(28)^ showed the efficacy and safety of 1 mg and 4 mg doses of intravitreal triamcinolone for vision loss related to macular edema due to branch RVO. The study concluded that while higher doses may offer better effectiveness, they also carry a higher risk of adverse effects. Therefore, adjusting intravitreal triamcinolone doses according to eye volume is important to optimize treatment results and reduce toxicity.

For retinal tamponade in vitrectomized eyes, medical gases serve as vitreous substitutes. The gases commonly used are sulfur hexafluoride (SF6) and perfluoropropane (C3F8) at nonexpansile, isovolumic concentrations-around 20% and 14%, respectively. When administered with syringes larger than 20 mL, these gases are safe and their effects predictable, regardless of the VCV. However, using these gases at low concentrations may cause the tamponade effect to last less than expected. Conversely, higher concentrations can cause a marked rise in IOP. Thus, when using undiluted gas, it is essential to carefully adjust the injected volume to achieve optimal results and prevent complications^(29)^.

This study has several important limitations that should be noted. It is a descriptive study relying on existing literature, and the proposed dosage table has not yet been tested in clinical trials to confirm possible differences in therapeutic outcomes. The suggested dosing framework is theoretical and intended as an initial guide for future prospective research. Only after such evaluations can these doses be validated for routine clinical use, which may also require adjustments to the dosages listed in package inserts. A cost-effective first step would be to reexamine key studies of these medications conducted in the last 20 years. In particular, for patients with available biometry data, this analysis could evaluate whether eye size affected treatment response. This reanalysis is critical to evaluate the practicality of the proposed dosage adjustments and their potential influence on treatment results.

To optimize intraocular drug concentration, it is essential to adjust intravitreal medications and gas doses based on AL and VCV. This strategy could lead to safer and more effective treatments. Nevertheless, these dosing adjustment proposals must be tested individually in clinical trials that take into account the specific properties of each medication or medical gas.

## Data Availability

The datasets generated during and/or analyzed during the current study are available in the manuscript.
